# Impact of the coverage of risk-reducing salpingo-oophorectomy by the national insurance system for women with *BRCA* pathogenic variants in Japan

**DOI:** 10.1038/s41598-023-28304-w

**Published:** 2023-01-19

**Authors:** Hidetaka Nomura, Akiko Abe, Atsushi Fusegi, Teruyuki Yoshimitsu, Satoki Misaka, Atsushi Murakami, Tsuyoshi Matsumoto, Shiho Tsumura, Motoko Kanno, Yoichi Aoki, Sachiho Netsu, Makiko Omi, Terumi Tanigawa, Sanshiro Okamoto, Kohei Omatsu, Mayu Yunokawa, Hiroyuki Kanao, Eri Habano, Hiromi Arakawa, Keika Kaneko, Arisa Ueki, Yurie Haruyama, Hitoshi Inari, Takayuki Ueno

**Affiliations:** 1 Department of Gynecology, Cancer Institute Hospital, Japanese Foundation for Cancer Research, Ariake 3-8-31, Koto-Ku, Tokyo, 135-8550 Japan; 2 Department of Clinical Genetics, Cancer Institute Hospital, Japanese Foundation for Cancer Research, Tokyo, Japan; 3 Breast Oncology Center, Cancer Institute Hospital, Japanese Foundation for Cancer Research, Tokyo, Japan

**Keywords:** Cancer, Medical research, Oncology

## Abstract

To determine the impact of the coverage of risk-reducing salpingo-oophorectomy (RRSO) and mastectomy (RRM) as well as genetic testing for *BRCA* pathogenic variants by the national insurance system in Japan. We compared the clinical background of women who underwent RRSO at our institution before and after its coverage by the national insurance system. Those who underwent RRSO between January 2017 and December 2019 and between April 2020 and March 2022 were classified as Period**.** A and B, respectively. Overall, 134 women underwent RRSO during the study period. In Period A and B, 45 and 89 women underwent RRSO for the study period was 36 and 24 months, respectively. Compared with Period A, the number of women who underwent RRSO per month increased by threefold in Period B (*p* < 0.01). In addition, the number of women who underwent surgery for breast cancer along with RRSO increased in Period B (*p* < 0.01). Although the number of women who underwent concurrent RRM with RRSO in Period B increased, the difference was not statistically significant. Compared with Period A, the number of women diagnosed with *BRCA* pathogenic variant increased by 3.9-fold, and the proportion of women who underwent concurrent hysterectomy at the time of RRSO decreased from 66 to 7.9% in Period B (*p* < 0.01). Owing to the introduction of the national insurance system, the number of women who underwent RRSO and concurrent surgery for breast cancer at the time of RRSO increased in Japan.

## Introduction

Women who inherited the pathogenic variants (PV) of the *BRCA1* or *BRCA2* (*BRCA1/2*) genes have increased risks of developing breast and ovarian cancers (OCs). Women in the general population have a 1.3% lifetime risk of developing OC, but this risk increases to 44% and 17% for women up to 80 years old with a *BRCA1* or *BRCA2* PV, respectively^1^. For women with *BRCA1/2* PV, risk-reducing salpingo-oophorectomy (RRSO) has been shown to decrease OC-specific mortality and overall mortality by approximately 80% and 70%, respectively^2–5^. According to the National Comprehensive Cancer Network’s (NCCN) guidelines^6^, women are generally recommended to undergo RRSO between 35 and 40 years old with *BRCA1/2* PV following completion of childbirth. Onset of OC in women with *BRCA2* PV occurs at an average of 8–10 years later than in those with *BRCA1* PV. As a result, in women with *BRCA2* PV, RRSO is reasonably delayed until 40–45 years of age. In Japan, RRSO for women with *BRCA1/2* PV was not covered by the national insurance system until March 2020. Hence, women who underwent RRSO before March 2020 paid for the procedure. Consequently, a previous study showed that only 31.4% of genetically confirmed women with *BRCA 1/2* PV in Japan underwent RRSO before March 2020^7^.

Since April 2020, RRSO and risk-reducing mastectomy (RRM) have been covered by the national insurance system in Japan^8^. Simultaneously, germline *BRCA* testing has also been covered by the national insurance system for the purpose of diagnosing hereditary breast cancer and OC (HBOC) in patients with breast cancer and/or OC and companion diagnostics for the use of poly ADP-ribose polymerase inhibitors. However, family members of women with HBOC but without a history of breast cancer and/or OC are not candidates for the national insurance system-covered RRSO even if they have *BRCA1/2* PV.

We have been performing RRSO for HBOC on women with *BRCA1/2* PV since 2011 as part of a clinical trial under the approval of the institutional review board^9^. In this study, we determined the impact of the coverage of RRSO by the national insurance system by comparing cases before and after the coverage date.

## Materials and methods

After approval from the institutional review board of the Cancer Institute Hospital (2010–1101), retrospective chart review was performed. Informed consent was obtained from all the participants treated at Cancer Institute Hospital, Tokyo, Japan. Cancer Institute Hospital had 686 beds and employed more than 2300 people to serve the patients. The study was conducted in accordance with the relevant guidelines and regulations of the institutional review board. Before the coverage of RRSO by the national insurance system, the inclusion criteria of women who wanted to undergo RRSO in our clinical study were as follows: (1) genetically confirmed germline *BRCA1/2* PV; (2) no desire to bear a child; (3) an understanding of the complications associated with RRSO and the possible symptoms of surgical menopause; (4) an understanding of the financial cost, which was not covered by the national insurance system; and (5) an understanding that RRSO is performed for clinical research. After the coverage of RRSO by the national insurance system, women with *BRCA1/2* PV who had a history of breast cancer (BC) could undergo RRSO through insurance coverage; however, women without a history of BC still needed to bear the expenses on their own to undergo RRSO. Since April 2020, RRSO has been covered by the national insurance system for those who have a history of BC with *BRCA1/2* PV in Japan. At the same time, *BRCA* testing was also covered for carriers with BC or OC. Furthermore, genetic testing for *BRCA* status has also been approved for patients with metastatic prostate and pancreatic cancer. Thus, even after April 2020, women who wanted to undergo RRSO but did not have BC were enrolled in our clinical study. Additionally, concurrent hysterectomy without medical indication is not covered by the national insurance system.

We compared the clinical background of women who underwent RRSO before and after its coverage by the Japanese national insurance system. Those who underwent RRSO between January 2017 and December 2019 and between April 2020 and March 2022 were classified as Period A and B, respectively. From January to March 2020, no one received RRSO because candidates had been informed that the insurance coverage for affected women would start from April 2020. And four women in Period B were those candidates.

Continuous variables were calculated as medians and compared in the Mann–Whitney U test. Categorical variables were compared in the Fisher’s exact test. Statistical analysis was performed using R software, version 3.0.1 (R Foundation for Statistical Computing, Vienna, Austria).

## Results

Overall, 134 women underwent RRSO at our institution during the study period. All procedures were performed laparoscopically. The baseline clinical characteristics of Period A and B are summarized in Table 1. In Period A, 45 women underwent RRSO in 36 months, whereas 89 women underwent RRSO in 24 months in Period B. The median age, *BRCA* status, personal BC history, and family history of OC within the third degree between the groups were not statistically different. In Period B, there were significantly more parous women. Four women who had been waiting for the introduction of national insurance underwent RRSO at the early stage of Period B.

**Table 1:** Background of women with *BRCA* pathogenic variants.

	Total	Period A		Period B		*p*-value
		*BRCA1*	*BRCA2*	*BRCA1*	*BRCA2*	
*BRCA* pathogenic variant carriers	134	45		89		** < 0.001**
		27 (60%)	18 (40%)	47 (52.8%)	42 (47.2%)	0.54
Median age at RRSO (range)	48.5 (38–74)	50 (38–74)		48 (32–73)		0.12
Personal breast cancer history	122	39 (86.7%)		83 (93.3)		0.35
Familial history of OC within third degree	53	16 (35.6%)		33 (37.1%)		1
Parous	99	28 (62.2%)		71 (79.8%)		**0.048**
Paid by insurance						
No	51	45 (100%)		6 (6.7%)		** < 0.01**
Yes	83	0		83 (93.3%)		
number per month		0		3.5		** < 0.01**

^a^Risk-reducing salpingo-oophorectomy.

^b^Breast cancer.

^c^Ovarian cancer.

The outcomes of the two groups are summarized in Table 2. The number of women who underwent RRSO per month increased by up to threefold in Period B (*p* < 0.01). Additionally, the number of women who underwent surgery for BC along with RRSO increased in Period B. The number of women who underwent concurrent RRM with RRSO in Period B also increased, but this was not statistically significant. One woman in Period A and three women in Period B had previously undergone hysterectomy for benign indications. Furthermore, the number of cases who underwent concurrent hysterectomy markedly decreased in Period B (*p* < 0.01). All women in Period A underwent hysterectomy, whereas five women in Period B underwent hysterectomy and paid out of pocket. Two women in Period B underwent concurrent hysterectomy that was covered by the national insurance system for abnormal cervical cytology and endometrial neoplasms, both of whose abnormalities were diagnosed at preoperative screening for RRSO. Both of these were revealed to be benign after a thorough pathological examination of the uterus. No surgical complications associated with concurrent hysterectomy occurred.

**Table 2:** Outcomes of risk-reducing salpingo-oophorectomy.

	Period A	Period B	*p*-value
Number	45	89	
Study period	36 months	24 months	
Number per month	1.25	3.71	
Unaffected carriers	Every 6 months	Every 4 months	0.74
Concurrent RRM^a^	4 (8.9%)	21 (23.6%)	0.07
Concurrent surgery for BC^b^	0	21 (23.6%)	** < 0.01**
Concurrent RRM and surgery for BC	0	7 (7.9%)	0.094
Concurrent hysterectomy (except previous hysterectomy)	29 (66%)	7 (7.9%)	** < 0.01**

^a^Risk-reducing mastectomy.

^b^Breast cancer.

Genetic testing was performed in 377 women in Period A and 1722 women in Period B in our hospital (Table 3). Compared with Period A, the number of genetic testings per month in Period B increased by 6.8-fold (71.8/10.5). The number of women in Period B diagnosed with *BRCA* PVs increased 3.9-fold (189/48) compared with those in Period A. Furthermore, there was a 5.9-fold (7.88/1.33) increase in the number of women with positive genetic testing per month in Period B.

**Table 3:** Genetic testing performed in each group.

	Period A		Period B	
	Number	Positive	Number	Positive
BRACAnalysis®⊠	245	25 (10.2%)	1720	188 (10.9%)
Breast cancer	180	14 (7.8%)	1288	110 (8.5%)
Ovarian cancer	65	11 (16.9%)	249	64 (25.7%)
Pancreatic cancer	0	0	112	8 (7.1%)
Prostate cancer	0	0	71	6 (8.5%)
Other genetic tests for HBOC^a^	132	23 (17.4%)	2	1 (50%)
Total	377	48 (12.7%)	1722	189 (11.0%)
Number per month	10.5	1.33	71.8	7.88

^a^Hereditary breast and ovarian cancer.

## Discussion

Our study revealed two important findings. After the coverage of RRSO by the Japanese national insurance system for women with *BRCA* PV, the number of women who underwent RRSO increased by threefold in Japan^10^. In addition, the number of women who underwent RRSO concurrently with the treatment for BC also increased.

Compared to the time when RRSO was not covered by the national insurance system, the number of women who underwent RRSO increased by threefold per month after its coverage. The increase in the number of women who underwent genetic testing also contributed to the increase in the number of cases that underwent RRSO because both were covered by the national insurance system. The number of women with positive genetic testing consequently increased by 5.9-fold. Therefore, there was a notable increase in the number of candidates for RRSO. Although, patients with pancreatic cancer and prostate cancer can receive genetic testing covered by the national insurance system for companion diagnosis for PARPi use, it is not allowed for diagnosis of HBOC, Keiran et al. reported that patients who are more financially capable, with insurance coverage, and diagnosed with BC and/or OC were more likely to undergo genetic testing^11^. Moreover, a higher income was associated with willingness to undergo testing and risk-reducing surgeries among cancer patients^12–14^. As shown in Table 3, the number of women in Period B diagnosed with *BRCA* PVs increased by 3.9-fold compared with those in Period A. In the future, unaffected family members with those probands will have opportunity to undergo RRSO. The women in Period B were not young enough to match the appropriate age derived from NCCN guidelines^6^. It was reported that women who underwent delayed RRSO were significantly older at the time of genetic testing than those who underwent timely RRSO^15^. To promote genetic testing for unaffected carriers, its coverage by the national insurance system for unaffected individuals is expected. Simultaneously, the fee for genetic testing, which is as high as $600 in Japan in contrast to less than $100 in South Korea, should be discounted. In Japan, genetic testing is expensive because it is outsourced, whereas this process can be performed in-house in South Korea^8^.

We had researched the psychosocial aspects of RRSO in our institution^16^. In the study, 16 women who underwent RRSO were interviewed before the surgery. The main reason to choose RRSO was “wishes of family members.’’ The reason why there were more parous women in Period B may be related with the results of the study. Specifically, wishes of their children might be one of the key factors for undergoing RRSO; family members’ wishes became easier to grant because of financial help. The decision to undergo RRSO depends on various factors, medical, physical, psychological and social contextual factors^17^. In this study, we found that one of the reasons for hesitancy to undergo RRSO was the economic problem in Japan. In general, Japanese women in their late 30 s to 40 s, which is the recommended age of undergoing RRSO, have no financial leeway because of childcare, home loan, and other expenses. The inclusion of RRSO under the national insurance system has resulted in adequate treatment for women with *BRCA* PV. On the other hand, it is reported that insurance coverage was not a barrier to receive genetic testing for unaffected but high-risk individuals^13^. Counseling efforts that are tailored to those individuals that emphasize health promotion and cancer prevention of *BRCA* testing are needed to improve testing rates.

The number of women who underwent concurrent hysterectomy at the time of RRSO decreased because the procedure was not covered by the national insurance system. In Period A, as much as 69% of women hoped and underwent hysterectomy. Considering the background of these women, an elevated risk of endometrial cancer was the most common indication. In 2016, the overall risk for uterine cancer after RRSO did not increase, whereas that for serous/serous-like endometrial cancer (EC) increased in women with *BRCA1* PVs^18^. In Jewish Israeli women, *BRCA1/2* PVs is associated with a 2.5 to 4 times increased risk for developing uterine cancer, especially serous papillary and sarcoma^19^. A multicenter study in The Netherlands concluded that women with *BRCA1/2* PVs have a 2- to threefold increased risk for EC, with the highest risk observed in women with rare subgroups of serous-like and p53-abnormal EC in mutation carriers^20^. In Japan, women with *BRCA2* PVs had a fourfold increased risk for EC^21^. Although there is no study that shows the positive impact on the overall survival of concurrent hysterectomy at the time of RRSO, this procedure must be discussed with each patient. Patients with early-stage EC have a favorable 5-year overall survival. However, those who have serous or serous-like tumors or stage IB or worse disease should undergo adjuvant chemotherapy in addition to pelvic and para-aortic lymphadenectomy^22^. Those treatments cause decreased quality of life even at an early stage. Considering the minimal complications associated with hysterectomy at RRSO, this procedure can be a treatment alternative to avoid EC. A recent study from Canada reported that 8.9% of women chose to undergo concurrent hysterectomy for fear of developing EC^23^. As there is no confirmed method to detect EC at its early stages, prophylactic hysterectomy can be an option for women with *BRCA1/2* PVs. Currently, in Japan, women who desire concurrent hysterectomy need to pay the fee for not only hysterectomy but also RRSO from their own pockets.

The number of women who underwent RRSO at the time of operation for BC increased as it was covered by the national insurance system. However, patients with BC who visited our hospital to discuss the concerns they had regarding different treatment options. For example, they discussed about whether they should undergo genetic testing, which procedure to choose (total mastectomy or partial mastectomy), whether to undergo RRM, and whether to undergo RRSO. Physicians treating BC, gynecologists, and genetic counselors are forced to respond to these patients’ requests as a team.

Our study had some limitations. First, this study was a single-center retrospective study. Therefore, it does not reflect the situation in Japan. Second, we could not analyze the clinical backgrounds of those who did not undergo RRSO. There are so many factors to hesitate RRSO, such as, family history, disease awareness, environment of individuals, social background, and economical problems. Hence, even after the introduction of the national insurance system for RRSO, there are still many women who do not undergo the surgery. Third, some of women who underwent RRSO at our hospital had been examined at other institutions. Hence, exact correlation of the increased number of positive genetic testing and increased number of RRSO currently remains unclear.

Conclusively, owing to the inclusion of the national insurance system for RRSO, RRM, and genetic testing for *BRCA1/2* PVs, a remarkable increase in the number of women who underwent RRSO in Japan was observed. Women who underwent concurrent treatment for BC and RRM also increased. To provide adequate treatment and management as per individuals’ requests, genetic testing for unaffected women and concurrent hysterectomy should be also covered by the national insurance system, and the fee for genetic testing should be discounted.

## Data Availability

The datasets used and analysed during the current study are available from the corresponding author on reasonable request.
